# Contribution of Each Leg to the Control of Unperturbed Bipedal Stance in Lower Limb Amputees: New Insights Using Entropy

**DOI:** 10.1371/journal.pone.0019661

**Published:** 2011-05-13

**Authors:** Petra Hlavackova, Céline Franco, Bruno Diot, Nicolas Vuillerme

**Affiliations:** 1 FRE 3405, AGIM (AGeing Imaging Modeling), CNRS-UJF-EPHE, Grenoble, France; 2 Faculty of Physical Culture, Palacky University, Olomouc, Czech Republic; 3 UJF-Grenoble 1/CNRS/TIMC-IMAG UMR 5525, Grenoble, France; 4 IDS SA, Montceau-les-Mines, France; 5 CIC-IT 805, INSERM/AP-HP, Raymond Poincaré Hospital, EA 4497, Garches, France; McMaster University, Canada

## Abstract

The present study was designed to assess the relative contribution of each leg to unperturbed bipedal posture in lower limb amputees. To achieve this goal, eight unilateral traumatic trans-femoral amputees (TFA) were asked to stand as still as possible on a plantar pressure data acquisition system with their eyes closed. Four dependent variables were computed to describe the subject's postural behavior: (1) body weight distribution, (2) amplitude, (3) velocity and (4) regularity of centre of foot pressure (CoP) trajectories under the amputated (A) leg and the non-amputated (NA) leg. Results showed a larger body weight distribution applied to the NA leg than to the A leg and a more regular CoP profiles (lower sample entropy values) with greater amplitude and velocity under the NA leg than under the A leg. Taken together, these findings suggest that the NA leg and the A leg do not equally contribute to the control of unperturbed bipedal posture in TFA. The observation that TFA do *actively* control unperturbed bipedal posture with their NA leg could be viewed as an adaptive process to the loss of the lower leg afferents and efferents because of the unilateral lower-limb amputation. From a methodological point of view, these results demonstrate the suitability of computing bilateral CoP trajectories regularity for the assessment of lateralized postural control under pathological conditions.

## Introduction

In recent years, a growing number of studies have been designed to explore the dynamical structure of the centre of foot pressure (CoP) trajectories in terms of entropy (regularity/predictability) to provide more insight into mechanisms involved in the control of bipedal unperturbed stance ([1], for a recent review). Roughly speaking, the more irregular/unpredictable the series, the greater entropy values [2], [3]. Interestingly, thanks to the computation of CoP entropy, specific postural behaviors induced either by age [4], health status [5]–[8], expertise [9], [10], postural task [11], sensory environment [4], [8], [10], [12] and cognitive context [7], [8], [10], [12], [13] have successfully been highlighted. Taken together, results of these studies have suggested that the regularity of CoP fluctuation could be considered as a marker of the amount of attention invested in the control of bipedal unperturbed posture (see [11], Figure 4), hence justifying the surplus value of CoP entropy measures in understanding postural control mechanisms. Computed from the bilateral CoP data, these measures, in complement to spatio-temporal conventional measures, have further proven their effectiveness and suitability in disentangling the relative contribution of the non-paretic and the paretic leg to the control of unperturbed bipedal posture after stroke [6]. Indeed, by showing a greater CoP regularity under the non-paretic foot than the paretic foot, these authors suggested that stroke patients actively control their unperturbed bipedal posture with their non-paretic leg. The present study was designed to further investigate the suitability of computing bilateral CoP trajectories regularity for the assessment of lateralized postural control in other individuals suffering from lateralized postural impairment. Among these pathological populations, persons with unilateral transfemoral amputation (TFA) are known to exhibit asymmetry in weight bearing [14]–[21] and in the patterns of plantar CoP displacements under their non-amputated (NA) and amputated (A) legs [17]–[20]. Surprisingly, the control of unperturbed bipedal stance has not been investigated through the analysis of the dynamical structure of the CoP in lower limb amputees yet. Considering the above-mentioned literature ([1] for a recent review), we believe that the recourse to CoP entropy measures could provide further relevant information about characterizing the control of unperturbed bipedal posture in TFA. Within this context, our purpose was to assess the relative contribution of each leg to unperturbed bipedal posture in TFA, through the use of the spatio-temporal (CoP amplitude 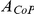 and CoP velocity 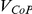) and dynamical (CoP regularity 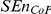) posturographic measures of CoP trajectories under each leg [6]. A greater contribution of the NA leg, yielding greater amplitude and velocity, accompanied by a more regular CoP profile under the NA leg than under the A leg, was expected.

## Results

**Figure 1:**
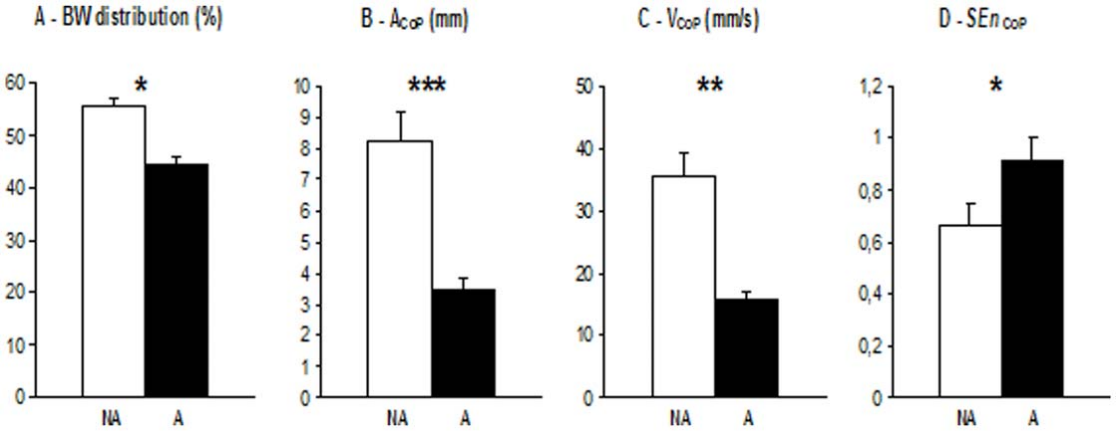
Mean and standard error of the mean body weight distribution (BW distribution) (panel A), the amplitude (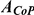) (panel B), the velocity (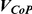) (panel C), and the regularity (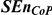) (panel D) of centre of foot pressure (CoP) trajectories under the non-amputated (NA) leg (white bars) and the amputated (A) leg (black bars). The P-values for comparisons between postural parameters computed from the NA leg and those computed from the A leg are reported (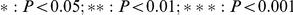).

As illustrated in Figure 1:

- BW distribution was significantly higher on the NA leg than on the A leg (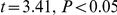, Fig. 1A);

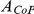
 was significantly higher the NA leg than on the A leg (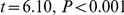, Fig. 1B);

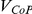
 was significantly higher on the NA leg than on the A leg (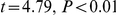, Fig. 1C);

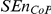
 was significantly lower on the NA leg than on the A leg (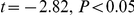, Fig. 1D).

## Discussion

By separately assessing the posturographic measures of each foot, the present study showed that the NA leg and the A leg do not equally contribute to the control of unperturbed bipedal posture in TFA. To be more precise, two kinds of asymmetry have been evidenced. The first asymmetry involves the weight bearing, with the observation of a larger BW distribution applied to the NA leg than to the A leg (Fig. 1A). This feature, in line with previous reports [14]–[21] has been suggested to arise from proprioceptive and cutaneous loss due to leg amputation. The second asymmetry concerns the dynamic process aimed at stabilizing unperturbed bipedal posture. In accordance with previous reports [17]–[20], larger 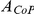 and 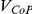 values were observed on the NA leg than on the A leg (Fig. 1B and 1C, respectively). Considering the functional significance of these two postural parameters [22], these results could reflect the relative ineffectiveness and the larger amount of neural control activity required by the TFA to regulate their CoP under their NA leg, respectively. From a clinical point of view, such a postural behavior has been suggested to reflect an adaptation to the loss of the lower leg afferents and efferents because of the unilateral lower-limb amputation [14], [15], [17]. The increase in CoP displacements observed under the NA leg relative to the A leg could hence reflect enhanced exploratory testing of the ground movements with sensors of the NA leg's feet aimed at providing supplementary somatosensory inputs to the central nervous system to preserve/facilitate postural control in condition of somatosensory loss from the amputated leg. Note that a similar postural response to unilateral neuromuscular perturbation has recently been observed in young healthy adults subjected to unilateral muscle fatigue localized at their ankle [23] and hip [24]. It has been suggested that subjects exhibited larger CoP displacements under their non-fatigued leg than under their fatigued leg during unperturbed bipedal standing to cope with an alteration of neuromuscular function induced by a fatiguing exercise unilaterally localized at their dominant leg's ankle [23] and hip muscles [24]. To our knowledge, no previous studies have investigated this possibility. Interestingly, recourse to the analysis of the observation of an asymmetry in the dynamical structure of bilateral CoP trajectories in terms of its regularity allowed to test this hypothesis, and, hence to provide further significant information about characterizing the control of unperturbed bipedal posture in TFA lends support to this assumption. Indeed, a greater CoP regularity/predictability was noticed under the NA leg than under the A leg, as indicated by the lower 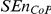 values reported on the NA leg than on the A leg (Fig. 1D). This result may seem surprising given that the common expectation is to find lower 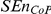 values for unhealthy or less adaptable to change systems [25]. As recently highlighted in the review of [1], the interpretation of entropy is not straightforward: entropy depends on automaticity, attention and noise. High values of entropy may be attributed to expert systems which need not pay attention to balance and are ready for the unexpected but also to unsustainable ones which are not able yet to exert an effective attentive control. This latter case may account for the greater value of entropy observed under the A leg. Moreover from an attention-control point of view, the lower 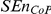 values reported on the NA leg suggests that TFA do actively control unperturbed bipedal posture with their NA leg. At this point, this postural behaviour could be considered as an adaptive process that could be common in other patient populations with lateralized disorders. In this respect, our results are in line with those recently obtained in stroke patients [6]. Interestingly, by using the same spatio-temporal (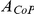 and 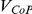) and dynamical (CoP regularity, 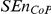) posturographic measures of bilateral CoP trajectories we computed in the present study, these authors have reported a greater relative contribution of the non-paretic foot to the control of unperturbed bipedal posture, characterized by a more regular CoP profile with greater amplitude and velocity under the non-paretic foot than under the paretic foot. Taken together, the above-mentioned results and ours evidence that the relative contribution of the non-affected and the affected leg to the control of unperturbed bipedal posture, quantified by the amplitude, velocity and regularity of the bilateral CoP trajectories, can be modified after a neurological (stroke) [6] or traumatic event (lower limb amputation) (the present study). From a methodological point of view, these results further demonstrate the suitability of computing bilateral CoP trajectories regularity for the assessment of lateralized postural control under pathological conditions. Within this context, to further investigate the generalization of these results, a future experiment involving patients with Parkinson's disease, who also have demonstrated asymmetry in postural control between their feet [26], is included in our immediate plans. Finally, it is important to mention that, considering the importance of foot position on CoP-based measures [27], [28], the experimental procedure used in the present study involved a standardized foot position. Along these lines, it is possible that this imposed upright posture (i.e., feet parallel and separated by the same distance for each participant) could have been quite different from a free and/usual bipedal posture for some participants and could have contributed to specific postural behaviour regarding the relative contribution of the NA and A legs to the control of unperturbed bipedal posture. This specific issue deserves further investigation which is included in our immediate plans.

## Materials and Methods

### Subjects

Eight unilateral trans-femoral amputees (TFA) (mean age: 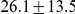 years; cause of amputation: 8 trauma; mean time since amputation: 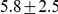 years) voluntarily participated in this study. The study was conducted in accordance with the Declaration of Helsinki and was approved by the national ethics committee (Socit Française des Technologies pour l'Autonomie et de Grontechnologie). Participants gave their informed written consent to the experimental procedure. Inclusion criteria were age 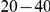 years, a unilateral traumatic trans-femoral amputation at least 1 year earlier, the use of a prosthesis on a daily basis, the ability to stand with a prosthesis without walking aids for at least 10 min and painless weight bearing on the prosthesis. Participants were excluded if they had any medical conditions that could affect their mobility or balance, such as neurological, orthopaedic or rheumatic disorders, the use of antipsychotic drugs, antidepressants or tranquilizers, otitis media. Additional exclusion criteria for TFA included reduced somatosensory sensibility of the non-amputated leg, ulceration or pain at the stump, or fitting problems of the prosthesis.

### Task and procedure

Subjects stood, eyes closed, on a plantar pressure data acquisition system (Zebris FDM-S Multifunction Force Measuring Plate system; sampling frequency: 100 Hz), with feet parallel separated by 10 cm, and their arms hanging loosely by their sides. This system includes capacitive force sensors arranged in matrix form (resolution: 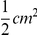, accuracy: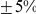) allowing the real-time acquisition of the force distribution under each foot and the computation of the instantaneous position of the CoP under each foot. Subject's task was to stand as still as possible during the trial. Three 30-s trials were performed. Rest periods of 60-s were provided between each trial during which subjects were allowed to sit down. The repeatability of foot placement between trials was ensured by outlining the feet on the top of the plantar pressure data acquisition system.

### Data analysis

As mentioned above, the plantar pressure data acquisition system used, allowed the simultaneous recording of the force distributions and the CoP trajectories under the NA leg and the A leg. Four dependent variables were then computed to describe the subject's postural behavior: (1) body weight (BW) distribution, (2) amplitude (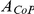), (3) velocity (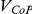) and (4) regularity (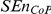) of centre of foot pressure (CoP) trajectories under the A leg and the NA leg. First the body weight distribution was determined by calculating the ratio between the force distribution under each leg and the total force exerted on the platform, the lot afterwards multiplied by 100 to obtain a percentage. Concerning the COP analysis, before calculation, the bilateral CoP data were mean-centered and denoted 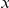 in the medio-lateral and 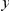 in the antero-posterior directions. Given that the effect of filtering on non-linear analysis is still debated, data were not filtered in this study [4], [29]. Then, the resultant distance time series denoted 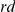 was calculated for each leg as follows [22]: 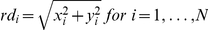, where 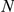 is the size of the sample. 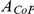, 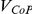 and 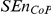 were then derived from the 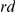 time series as recently described by [6].

On the one hand, to quantify the amount of postural sway under each leg, the root mean square of the CoP displacements (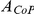 in 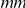) and velocities (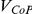 in 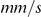) were calculated as follows: 
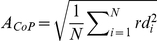
, 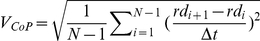 where 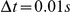 On the other hand, to give insights into the dynamical structure of the CoP trajectories under each leg in terms of regularity, their respective sample entropy was calculated. To make this measure size and scale-independent their 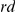 time series were mean-centered and normalized to unit variance before calculation. Sample Entropy reflects the amount of regularity or predictability of a time series by quantifying to what extent a pattern is likely to repeat more or less within a time series. More precisely it is the negative natural logarithm of the conditional probability that two datasets of 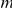 points similar within an error tolerance of 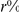 of the standard deviation of the time series remain similar at the next point 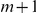, excluding self-matches. Note that 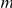 and 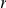 are the embedded parameters of this non-linear analysis. Their establishment follows an optimization procedure based on a metric of the efficiency of the entropy estimate which led in our case to select the couple (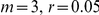). For details about the calculation of Sample entropy and the optimization procedure of the parameters 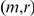, see [30], [31]. 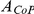, 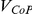 and 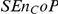 have been related to the effectiveness of the postural control system, the amount of postural regulatory activity [22] and to the amount of attention invested in postural control, respectively [11], [12]. Note that these postural parameters have been recently used for the evaluation of contribution of each leg to the control of unperturbed bipedal posture after stroke [6].

### Statistical analysis

The means of the three postural measurements recorded were used for statistical analyses. BW distribution, 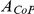, 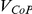 and 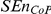 of the NA leg were compared with the A leg through a paired t-tests. The level of significance was set at 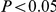.
